# Structure-Activity Relationship Analysis of Benzotriazine Analogues as HIV-1 Latency-Reversing Agents

**DOI:** 10.1128/AAC.00888-20

**Published:** 2020-07-22

**Authors:** Eric S. Sorensen, Amanda B. Macedo, Rachel S. Resop, J. Natalie Howard, Racheal Nell, Indra Sarabia, Daniel Newman, Yanqin Ren, R. Brad Jones, Vicente Planelles, Adam M. Spivak, Alberto Bosque

**Affiliations:** a Department of Microbiology, Immunology, and Tropical Medicine, George Washington University, Washington, DC, USA; b Department of Medicine, Division of Infectious Diseases, University of Utah, Salt Lake City, Utah, USA; c Infectious Disease Division, Weill Cornell Medical College, New York, New York, USA; d Department of Pathology, Division of Microbiology and Immunology, University of Utah School of Medicine, Salt Lake City, Utah, USA

**Keywords:** HIV reservoir, LRA, human immunodeficiency virus, latency reversal agent, shock and kill

## Abstract

“Shock and kill” therapeutic strategies toward HIV eradication are based on the transcriptional activation of latent HIV with a latency-reversing agent (LRA) and the consequent killing of the reactivated cell by either the cytopathic effect of HIV or an arm of the immune system. We have recently found several benzotriazole and benzotriazine analogues that have the ability to reactivate latent HIV by inhibiting signal transducer and activator of transcription 5 (STAT5) SUMOylation and promoting STAT5 binding to the HIV long terminal repeat and increasing its transcriptional activity. To understand the essential structural groups required for biological activity of these molecules, we performed a systematic analysis of >40 analogues. First, we characterized the essential motifs within these molecules that are required for their biological activity. Second, we identified three benzotriazine analogues with similar activity. We demonstrated that these three compounds are able to increase STAT5 phosphorylation and transcriptional activity. All active analogues reactivate latent HIV in a primary cell model of latency and enhance the ability of interleukin-15 to reactivate latent HIV in cells isolated from aviremic participants. Third, this family of compounds also promote immune effector functions *in vitro* in the absence of toxicity or global immune activation. Finally, initial studies in mice suggest lack of acute toxicity *in vivo*. A better understanding of the biological activity of these compounds will help in the design of improved LRAs that work via inhibition of STAT5 SUMOylation.

## Abstract (precis)

“Shock and kill” therapeutic strategies toward HIV eradication are based on the transcriptional activation of latent HIV with a latency-reversing agent (LRA) and the consequent killing of the reactivated cell by either the cytopathic effect of HIV or an arm of the immune system. We have recently found several benzotriazole and benzotriazine analogues that have the ability to reactivate latent HIV by inhibiting signal transducer and activator of transcription 5 (STAT5) SUMOylation and promoting STAT5 binding to the HIV long terminal repeat and increasing its transcriptional activity.

## INTRODUCTION

The presence of an HIV latent reservoir precludes viral eradication from people living with HIV (PLWH) (1–5). The development of strategies aimed toward HIV eradication have mostly focused on the development of latency-reversing agents (LRAs) that transcriptionally reactivate latent HIV (6). As of today, several classes of LRAs with a myriad of mechanisms of action have been developed (for a review, see reference 7). Among the different classes of LRAs, we recently discovered a new family of compounds that reactivate latent HIV by targeting signal transducer and activator of transcription 5 (STAT5) SUMOylation (8). These compounds block SUMOylation of phosphorylated STAT5 and increase the nuclear presence, as well as the long-terminal repeat (LTR) binding ability of STAT5. This increase led to enhanced viral transcription and reactivation from latency both in a primary cell model of HIV latency and in CD4 T cells isolated from aviremic participants (8). These compounds represent a novel class of molecules classified as STAT SUMOylation inhibitors and are the first of this class. Further development of these compounds is warranted in order to fully characterize their potential use for HIV cure approaches.

This group of compounds has historically been used as reagents for laboratory methods of peptide synthesis. In that context, these compounds function as inhibitors of racemization to avoid racemic mixtures (8, 9), but no biological activity was ascribed to them. In the present study, we performed a systematic analysis of the LRA and STAT SUMOylation inhibitor activity of >40 analogues with similar chemical structures or activities as racemization inhibitors. Our results indicate that their biological activity as STAT SUMOylation inhibitors is independent of their *in vitro* activity as racemization inhibitors. Furthermore, we used different cellular models to ascertain the structural components required for their biological activity, including their ability to reactivate latent HIV in cells isolated from aviremic participants. Using mice, we evaluated their acute toxicity, as well as their pharmacodynamics. We found lack of toxicity and signs of systemic inflammation. Our results indicate that targeting STAT SUMOylation with benzotriazine analogues may be a suitable pharmacological strategy to target the latent HIV reservoir.

## RESULTS

### Novel benzotriazine analogues with biological activity.

We have previously characterized two families of compounds, benzotriazole and benzotriazine analogues, with the ability to reactivate latent HIV in primary CD4 T cells (8). These compounds reactivate latent HIV by inhibiting STAT5 SUMOylation, increasing STAT5 transcriptional activity and binding of STAT5 to the HIV LTR (8). In this previous study, 3-hydroxy-1,2,3-benzotriazin-4(3*H*)-one (HODHBt) was the compound exhibiting the highest activity.

**FIG 1:**
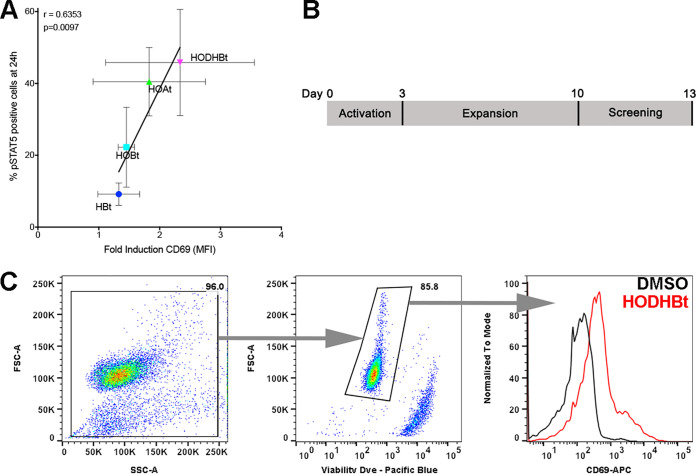
The activity of STAT SUMOylation inhibitors correlates with the surface induction of CD69. (A) The levels of pSTAT5-positive cells correlated with the induction of CD69. Samples were grouped by treatment. Error bars indicate the standard deviations (SD). Correlations and *P* values were calculated using all 20 data points. (B) Schematic of cellular screening of benzotriazine activity in primary CD4 T cells. (C) Representative flow cytometry analysis.

**FIG 2:**
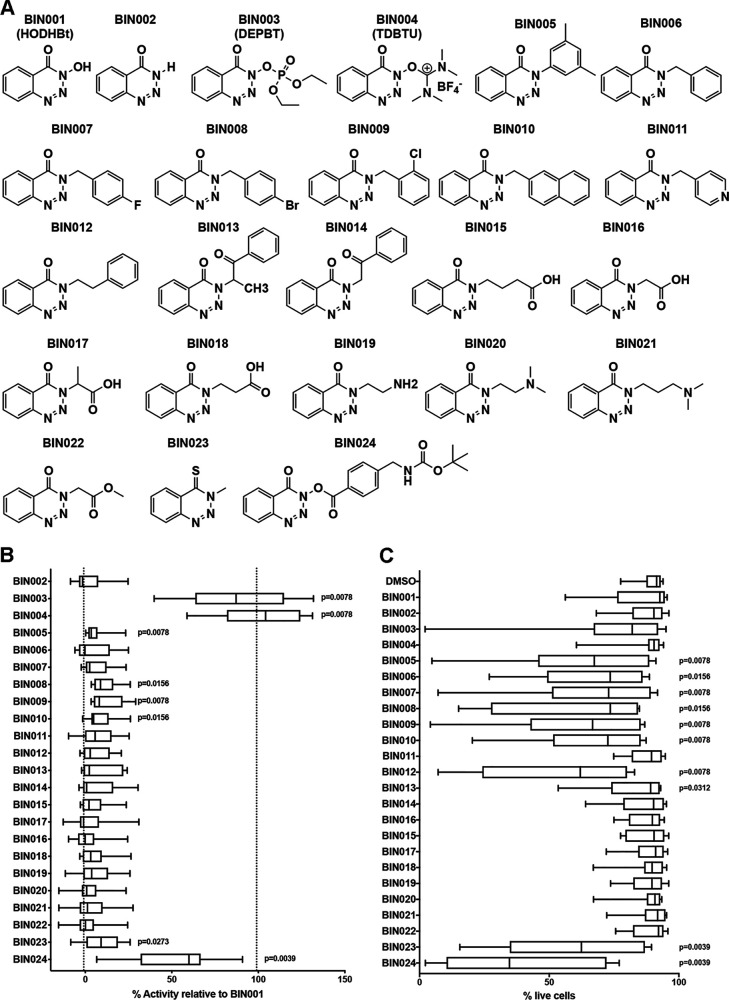
Screening for biological activity of benzotriazine derivates. (A) Structure of 24 commercially available benzotriazine analogues. (B) Analysis of activity of the different analogues relative to BIN001 (HODHBt), the derivative with the highest LRA activity, in nine different donors. Error bars indicate maximum and minimum values, and a Wilcoxon signed rank test was used to calculate *P* values. (C) Analysis of toxicity of the different analogues. Error bars indicate maximum and minimum values. A nonparametric Wilcoxon matched-pair signed-rank test relative to the DMSO control was used to calculate *P* values.

We wanted to understand the structure-activity relationships (SAR) of this family of compounds as STAT SUMOylation inhibitors. We have previously shown that the activity of these compounds correlated with their ability to maintain phosphorylation of STAT5 (8). These compounds are also able to increase expression of CD69 on the surface of CD4 T cells albeit to a much lesser degree compared to antigen stimulation (2.6- versus 19.5-fold) (8). The slight induction of CD69 correlated with the ability of these compounds to increase STAT5 phosphorylation (Fig. 1A). With that in mind, we designed a screening method using human primary CD4 T cells to identify other analogues with potential LRA activity and no toxicity *in vitro* using CD69 as a biomarker. First, naive CD4 T cells were isolated from HIV-negative donors and activated with beads coated with αCD3/αCD28 for 3 days (Fig. 1B). Activated cells were expanded in the presence of interleukin-2 (IL-2) for 7 additional days (Fig. 1B). At day 10, cells were cultured in the presence of IL-2 and a benzotriazine derivative. Three days later, the cells were stained with an anti-CD69 antibody and a viability dye and analyzed by flow cytometry (Fig. 1C). With this screening methodology, we first screened a panel of 23 commercially available benzotriazine analogues (labeled BIN) (Fig. 2A and see Data Set S1 in the supplemental material). Initially, all the compounds were tested at 100 μM in cells from nine donors (five male and four female). This concentration was chosen based on the maximal activity of the original compound HODHBt (8). The results were normalized to the activity of our original compound HODHBt (BIN001) versus a dimethyl sulfoxide (DMSO) control. Of the 23 compounds tested, 8 had detectable activities. Compounds BIN003 (DEPBT) and BIN004 (TDBTU) showed activities similar to that of BIN001 (Fig. 2B) with no *in vitro* toxicity (Fig. 2C). BIN0024 had partial activity (53% ± 27.5%) but was toxic *in vitro* (Fig. 2B and C). BIN005, BIN008, BIN009, BIN010, and BIN023 retained less than 10% of the mean activity compared to BIN001 but presented some toxicity *in vitro* (Fig. 2B and C).

**FIG 3:**
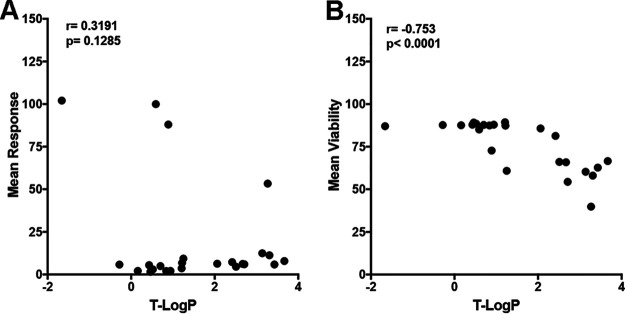
Relationship between the activity, toxicity, and hydrophobicity of benzotriazine analogs. (A and B) Spearman correlation of the theoretical *n*-octanol-water partition coefficient (T-logP) of each compound with the normalized response (A) or viability (B).

To begin to understand SAR of these compounds, we addressed whether their hydrophobicity influenced their biological activity. To that end, the theoretical *n*-octanol-water partition coefficient (T-logP) of each compound was calculated (https://www.molinspiration.com/cgi-bin/properties) (Data Set S1). Although biological activity does not seem to be correlated with the hydrophobicity of each derivative (Fig. 3A), toxicity and hydrophobicity are highly correlated: the higher the hydrophobicity of the compound, the higher its toxicity in primary CD4 T cells (Fig. 3B).

In our previous study, we also identified two benzotriazole analogues (labeled BOL) with LRA activity (8). We then performed a similar study to identify additional benzotriazoles with biological activity (Fig. S1A and Data Set S1). In our previous study, 1-hydroxybenzotriazole (HOBT; BOL001) was a weaker LRA than BIN001. The activity of this compound in our screening was also lower compared to BIN001 (Fig. S1B). None of the other 13 benzotriazole analogues tested had increased activity, and some of them showed toxicity *in vitro* (Fig. S1B and S1C). The second compound previously published was the azabenzotriazole derivative 1-hydroxy-7-azabenzotraziole (HOAT; BOL015). This compound had higher activity than BOL001 but lower than BIN001. All the other azabenzotriazole analogues tested (BOL016 to BOL019) had similar activity to BOL015, higher than BOL001 but lower than BIN001 with no overt toxicity *in vitro* (Fig. S1B and C).

Most of these compounds are peptide coupling reagents commonly used during peptide synthesis *in vitro* as racemization inhibitors (9). To address whether their racemization inhibitory activity was required for their biological activity, we tested a panel of five structurally unrelated peptide coupling reagents (labeled “PCRs” in Fig. S2A). None of the compounds tested had substantial activity (Fig. S2B and C). In conclusion, we identified several analogues with activity similar to our previously reported compounds and demonstrated that their biological activity is independent of their previously described activity as racemization inhibitors.

### Sustained STAT5 phosphorylation by benzotriazine analogues.

**FIG 4:**
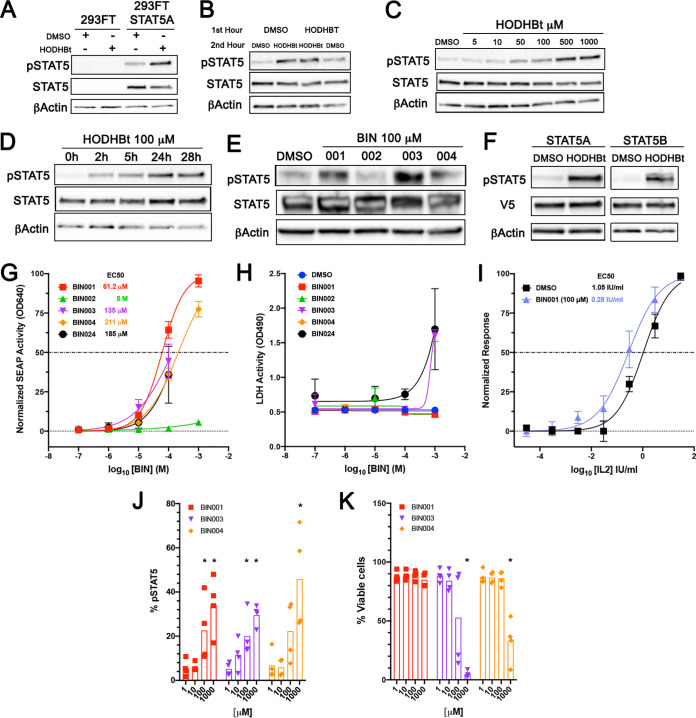
Ability of new benzotriazine analogues to increase STAT5 phosphorylation. (A) Analysis of the levels of pSTAT5 and STAT5 after treatment with 100 μM HODHBt in 293FT cells and 293FT-STAT5A. (B) Analysis of the levels of pSTAT5 and STAT5 after treatment with either DMSO or 100 μM HODHBt for 1 h, followed by a second hour incubation with either 100 μM HODHBt or DMSO. (C) Analysis of the levels of pSTAT5 and STAT5 after treatment of 293FT-STAT5A with increased concentrations of HODHBt. (D) Analysis of the levels of pSTAT5 and STAT5 after treatment of 293FT-STAT5A with 100 μM HODHBt at different times. (E) Analysis of the levels of pSTAT5 and STAT5 after treatment of 293FT-STAT5A with 100 μM concentrations of different benzotriazine analogues. (F) Analysis of the levels of pSTAT5 and STAT5 after transfection of either STAT5A or STAT5B in 293FT cells and treatment with either DMSO or 100 μM HODHBt. (G) Dose response of STAT5 activity of benzotriazine analogues ranging from 1 μM to 1 mM in HEK-Blue IL-2 cells. The data represent the means ± the SD of an experiment performed in sextuplets. (H) Toxicity of benzotriazine analogues ranging from 1 μM to 1 mM in HEK-Blue IL-2 cells. The data represent the means ± the SD of an experiment performed in sextuplets. (I) Dose response of STAT5 activity of IL-2 in the presence of 100 μM BIN001 in HEK-Blue IL-2 cells. The data represent the means ± the SD of an experiment performed in sextuplets. (J) Levels of STAT5 phosphorylation in CD4T cells from four donors treated with IL-2 in the presence of the indicated benzotriazine derivative at concentrations ranging from 1 μM to 1 mM. (K) Levels of toxicity in four donors treated with IL-2 in the presence of the indicated benzotriazine derivative at concentrations ranging from 1 μM to 1 mM. A Mann-Whitney test was used to calculate *P* values.

We have previously shown that HODHBt and related compounds work by decreasing STAT5 SUMOylation with a concomitant increase in the levels of STAT5 phosphorylation in human primary CD4 T cells (8). In order to further characterize these compounds, we stably transduced 293FT cells with a lentiviral vector expressing STAT5A (see Materials and Methods). 293FT-STAT5A cells expressed detectable levels of STAT5A compared with the parental cell line (Fig. 4A). HODHBt, but not DMSO, increased the levels of STAT5A phosphorylation (pSTAT5A) while maintaining constant levels of total STAT5A (Fig. 4A). We then addressed whether their ability to maintain STAT5 phosphorylation is reversible or irreversible. Cells were treated with DMSO or HODHBt for 1 h. After 1 h, the medium was replaced with either media containing DMSO or HODHBt. The continuous presence of HODHBt maintained the levels of pSTAT5A, while replacing HODHBt with DMSO reduced the levels of pSTAT5A to background levels (Fig. 4B). This result demonstrates that the activity of these compounds is reversible.

Next, we performed a dose-response analysis. 293FT-STAT5A cells were incubated with increasing concentrations of HODHBt, and the levels of pSTAT5A were analyzed by Western blotting. As shown in Fig. 4C, HODHBt increased STAT5 phosphorylation in a dose-dependent manner. Next, we addressed the stability of these compounds *in vitro*. Cells were treated with HODHBt at different time points. HODHBT enhanced STAT5 phosphorylation as early as 2 h, and the levels of phosphorylation increased over time up to 28 h (Fig. 4D). Because the activity of HODHBt was reversible (Fig. 4B), this result suggests that HODHBt was stable *in vitro* and was not degraded for up to 28 h.

Next, we tested the ability of the new benzotriazine analogues to increase pSTAT5A levels. 293FT-STAT5A cells were treated with HODHBt (BIN001), BIN002, BIN003, and BIN004. As expected, BIN002 did not increase the levels of pSTAT5A, whereas both BIN003 and BIN004 increased the levels of pSTAT5 to an extent similar to that achieved by HODHBt (Fig. 4E). STAT5A and STAT5B are two paralog genes that share 96% protein homology, and there is no antibody that specifically recognizes phosphorylated Y694 STAT5A or Y699 STAT5B (10). To address whether HODHBt enhanced the phosphorylation of STAT5A, STAT5B, or both, we transfected 293FT cells with either STAT5A or STAT5B and analyzed the levels of STAT phosphorylation after treatment with either HODHBt or DMSO control. As shown in Fig. 4F, HODHBt increased the levels of phosphorylation of both STATs. To further evaluate whether the increase in phosphorylation is associated with an increase in STAT5 transcriptional activity, we used HEK-Blue IL-2 cells. This cell line has been specifically designed to monitor the activation of the JAK-STAT pathway by human IL-2, and it contains a STAT5-inducible secreted embryonic alkaline phosphatase (SEAP) reporter gene. We tested the ability of the different benzotriazine analogues to increase STAT5 transcriptional activity. Besides BIN002, all of the other analogues activate STAT5 in a dose-dependent manner, with BIN001 having the lowest 50% effective concentration (EC_50_) (Fig. 4G). In this system, both BIN003 and BIN024 had some toxicity at the highest concentration tested (Fig. 4H). This HEK293 cell line has been engineered to be responsive to IL-2. We then decided to test whether these compounds will increase IL-2-mediated STAT5 activation. As shown (Fig. 4I), BIN001 enhanced the ability of IL-2 to activate STAT5 transcriptional activity almost 4-fold. Finally, we decided to evaluate whether the new identified benzotriazine analogues could increase the phosphorylation of STAT5 in primary human CD4T cells. As previously reported for BIN001 (8), BIN003 and BIN004 also increase IL-2-mediated STAT5 phosphorylation in primary CD4T cells (Fig. 4J). Both BIN003 and BIN004 had some toxicity at the highest concentration tested in primary CD4T cells (Fig. 4K). Altogether, these results indicate that the activity of these compounds is reversible, dose dependent, and stable *in vitro* and further confirm their ability to increase the phosphorylation and transcriptional activity of STAT5.

### Evaluation of new benzotriazine analogues as LRAs.

**FIG 5:**
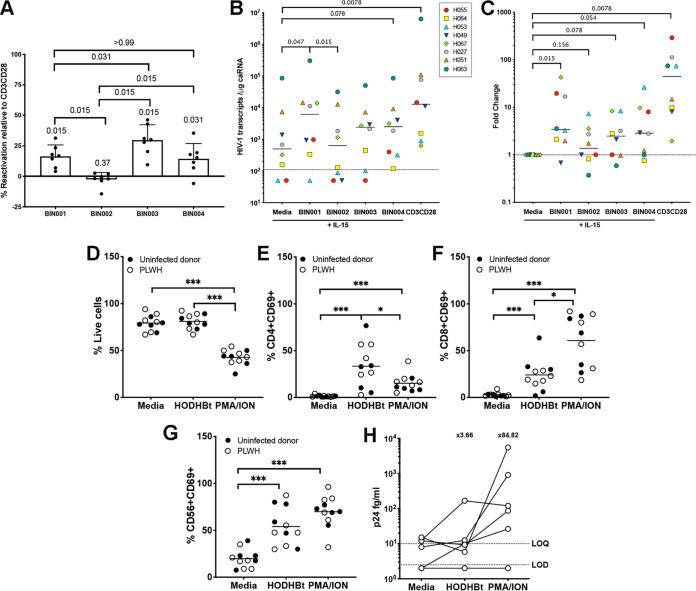
Ability of new benzotriazine analogues to reactivate latent HIV. (A) Reactivation of latent HIV in the T_CM_ model induced by 100 μM BIN001 (HODHBt), BIN002, BIN003, or BIN004. Values were normalized relative to CD3CD28 (*n* = 7), and a Wilcoxon signed-rank test was used to calculate *P* values. For all comparisons, *P* values were calculated by using a two-tailed Wilcoxon matched-pair signed-rank test. (B) Viral reactivation was measured in cells isolated from eight aviremic patients using the REVEAL assay treated with IL-15 in the presence of a 100 μM concentration of the indicated benzotriazine. For all comparisons, *P* values were calculated by using a two-tailed Wilcoxon’s matched-pair signed-rank test. (C) Fold change in HIV transcripts after reactivation with each of the benzotriazines relative to the medium control. A Wilcoxon signed-rank test was used to calculate the *P* values. (D) Percentages of live cells after 48 h of incubation of PBMCs with 100 μM HODHBt or PMA plus ionomycin (PMA/ION). Empty circles represent uninfected donors, and black circles represent ART-suppressed PLWH. For simplicity, the results were combined, and a unique mean is depicted (*n* = 5 to 6 each group). (E to G) CD69 induction on CD4 T cells (E), CD8T cells (F), and NK cells (G) (*n* = 5 to 6 each group). (H) p24 Gag protein was quantified by using a digital ELISA (*n* = 6) in cell culture supernatants from PBMCs isolated from ART-suppressed PLWH and treated with 100 μM HODHBt or PMA plus ionomycin (PMA/ION) for 4 days. A nonparametric Wilcoxon matched-pair signed-rank test was used to calculate *P* values.

To further evaluate the LRA activity of these newly characterized benzotriazine analogues, we used the cultured T_CM_ model of HIV latency (11, 12). Latently infected cells generated from seven donors were treated for 48 h with each of the analogues at 100 μM in the presence of IL-2. Viral reactivation was measured by flow cytometry and normalized to that of the positive control, anti-CD3/CD28 stimulation (see Materials and Methods). As with our previous analysis, BIN002 did not have LRA activity, whereas BIN003 and BIN004 did have LRA activity similar to that of HODHBt (BIN001) (Fig. 5A).

We have previously shown that HODHBt increases viral reactivation mediated by IL-2 in CD4T cells isolated from aviremic HIV-infected participants (8). We wanted to address whether these compounds also enhance viral reactivation mediated by other γc-cytokines. In particular, we were interested in IL-15. IL-15 and the IL-15 superagonist (IL-15SA) have been shown to reactivate latent HIV *in vitro* (13). IL-15SA ALT-803 (or N-803) is being currently tested in clinical trials for HIV eradication purposes (NCT02191098; https://clinicaltrials.gov/ct2/show/NCT02191098). We therefore tested whether these compounds can enhance IL-15 induced reactivation of latent HIV in cells isolated from aviremic participants. Resting CD4 T cells from eight participants were isolated and treated for 96 h with each of the analogues at 100 μM in the presence of IL-15. HIV cell-associated RNA was evaluated by quantitative PCR (qPCR) to evaluate whether each derivative increased HIV transcription. As expected, BIN001 (HODHBt) increased viral transcription compared to either IL-15 alone (media) or BIN002 (Fig. 5B). The other two BIN analogues tested also showed modest viral reactivation compared to media or BIN002, but it did not reach statistical significance (Fig. 5B). To evaluate the relative potency of each derivative, we calculated HIV mRNA fold induction relative to the medium control. Of all the analogues tested, BIN001 (HODHBt) had the highest potency, reactivating latent HIV with 3.4-fold HIV transcription versus the medium control. BIN004 increased viral transcription 2.84-fold, while BIN003 increased viral transcription 2.46-fold (Fig. 5C). On the other hand, the inactive compound BIN002 only increased HIV transcription 1.37-fold. Compared to maximal stimulation (CD3CD28), BIN001 (HODHBt) was 13-fold less efficient in inducing viral transcription. This agrees to our previously reported activity of HODHBt (8). In conclusion, we demonstrated that benzotriazine analogues that retain the ability to maintain STAT5 phosphorylated also maintain the ability to reactivate latent HIV both in a primary cell model of latency and in cells isolated from aviremic participants.

### Ability of HODHBt to promote immune effector functions.

Several current LRAs under clinical investigation, including the TLR7 agonist GS-9620 and IL-15, have been shown to enhance CD8T and NK cell activation that promotes HIV-specific immune responses (14–17). To further characterize whether this family of compounds could also have immunomodulatory properties, we treated peripheral blood mononuclear cells (PBMCs) from either uninfected donors or antiretroviral therapy (ART)-suppressed PLWH with HODHBt in the presence of ART for 4 days. At 48 h, we assessed global toxicity, as well as the induction of the activation marker CD69 in CD4T, CD8T, and NK cells. First, we did not observe any global toxicity when PBMCs were treated with HODHBt (Fig. 5D). In contrast, the positive control for T cell activation phorbol myristate acetate (PMA)/ionomycin severely decreased viability (Fig. 5D). HODHBt induced the expression of CD69 in CD4T (Fig. 5E), CD8T (Fig. 5F), and NK (Fig. 5G). We then addressed whether HODHBt alone was also able to reactivate latent HIV in PBMCs isolated from aviremic participants. We collected the supernatants from HODHBt-treated PBMCs of six ART-suppressed PLWH and measured viral p24Gag using the SIMOA p24 digital enzyme-linked immunosorbent assay (ELISA) at 4 days postactivation (8, 18). We were able to detect p24 antigen in supernatants from cells stimulated with HODHBt above the medium control in 3 of 6 participants, with an average fold induction of 3.66. In contrast, PMA/ionomycin induced p24 in 5 of 6 participants, with an average fold induction of 84.82 (Fig. 5H).

Finally, we investigated whether this family of compounds could induce global immune activation by measuring a panel of 14 cytokines released by PBMCs from uninfected donors and PLWH using a cytometric bead-based multiplex system and ELISA. HODHBt did not increase cytokine release versus the medium control for the different cytokines tested (Fig. S3). In conclusion, HODHBt can reactivate latent HIV and also has immunomodulatory properties in the absence of global immune activation.

### *In vivo* acute toxicity of benzotriazine analogues.

**FIG 6:**
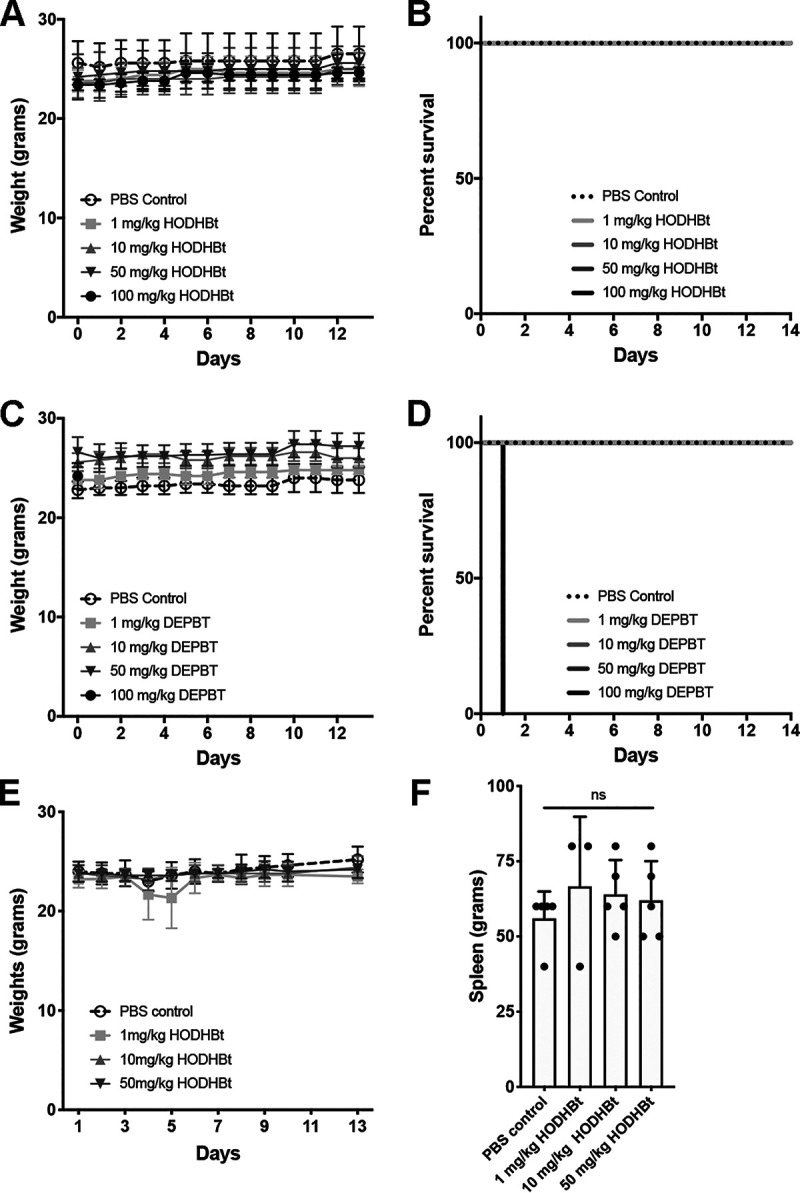
*In vivo* acute toxicity of benzotriazine analogues. (A) Total body weights of five mice before and after the s.c. administration of different doses of HODHBt. (B) Survival curves of mice injected with HODHBt. (C) Total body weights of mice before and after the s.c. administration of different doses of DEPBT. (D) Survival curves from mice injected with DEPBT. (E) Total body weights of mice before and after the i.p. administration of different doses of HODHBt. (F) Spleen weights of mice at day 13 before the i.p. administration of different doses of HODHBt. An unpaired *t* test was used to calculate *P* values.

There is no information on the safety of benzotriazine derivative administration *in vivo*. We therefore decided to perform toxicity test from a single exposure to evaluate what is the maximal tolerated dose of benzotriazine analogues in C57BL/6 mice. In particular, we focused on HODHBt and DEPBT (BIN003). Groups of five animals were challenged with increasing concentrations of both compounds by subcutaneous (s.c.) injection. Survival and weight were monitored up to 14 days postinjection. All of the animals injected with HODHBt survived and maintained their weight (Fig. 6A and B). On the other hand, DEPBT had acute toxicity at the highest concentration tested of 100 mg/kg in agreement with the toxicity observed at 1 mM in the *in vitro* systems (Fig. 6C and D).

To test for toxicity and activity via a different route of administration, groups of five animals were challenged intraperitoneally (i.p.) with increasing concentrations of HODHBt, and the weight was monitored for up to 13 days postinjection. There was no significant weight loss under any of the conditions tested (Fig. 6E). Finally, after 13 days, the animals were euthanized, and the size of each spleen was measured to evaluate potential splenomegaly. HODHBt did not increase the spleen size at any of the concentrations tested (Fig. 6F).

To initially test the pharmacodynamics of HODHBt, blood and splenocytes were collected 48 h after i.p. injection to measure levels of STAT5 phosphorylation, as well as the induction of CD69 in CD4 T cells. We could detect a trend toward increasing levels of pSTAT5 in circulating lymphocytes but not in splenocytes of mice treated with HODHBt (Fig. S4A). We did not detect an increase in CD69 in CD4 from either blood or splenocytes (Fig. S4B) or secretion or IL-6 (Fig. S4C). In conclusion, HODHBt was tolerated *in vivo* to concentrations up to 100 mg/kg, while DEPBT was tolerated *in vivo* at 50 mg/kg. Further studies are warranted to evaluate the pharmacodynamics and pharmacokinetics of this class of compounds.

## DISCUSSION

We have previously shown that inhibiting STAT5 SUMOylation may be a potential new target toward HIV eradication strategies (8). We previously found three compounds, two benzotriazole analogues and one benzotriazine derivative, that reactivated latent HIV in primary cells. These compounds are classified as STAT SUMOylation inhibitors. In the present study, we performed a comprehensive SAR analysis of these new classes of inhibitors.

**FIG 7:**
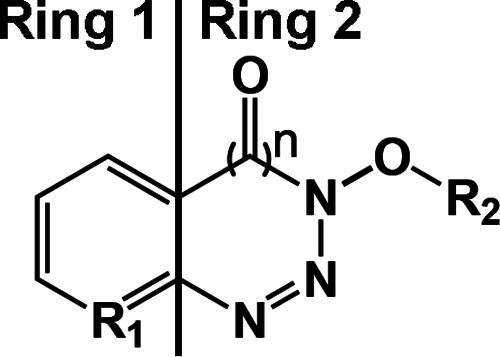
SAR of STAT SUMOylation inhibitors. A structure-activity relationship (SAR) map of benzotriazine analogues as LRAs is shown. A total of 43 structures were analyzed to generate this map.

Based on our results, we identified several structural components of these compounds that are either essential or important for their biological activity. All of these compounds consist of two main rings (Fig. 7). Ring 1 is a benzene group shared between both benzotriazine and benzotriazole analogues which is involved in their activity. Interestingly, substitution of the benzene group (R_1_═C) for a pyridine group (R_1_═N) increased activity of the resulting compounds (Fig. S1). Ring 2 can be either a triazole ring (*n* = 0) or a triazin-4(3*H*)-one (*n* = 1), with the latter having increased activity. The presence of a hydroxyl or other chemical group containing an oxygen in R_2_ is essential for activity. Furthermore, our studies also demonstrate that the biological activity of these compounds is independent of their previously known activity as racemization inhibitors.

The potential target of these compounds is still unknown. We have previously shown that these compounds inhibit STAT SUMOylation, but whether they are targeting any specific protein involved in this process is under investigation. SUMOylation is a posttranslational modification akin to ubiquitination. Protein SUMOylation involves an orchestrated process regulated by different proteins including SUMO-specific proteases (SENP1 to -3 and SENP5 to -7), SUMO-activating enzymes E1 (SAE1 and UBA2), a SUMO-conjugating enzyme E2 (UBC9), and a few SUMO E3 ligases (19). The major role of SUMOylation is to control localization of proteins within the cell (19). There are four SUMO isoforms expressed in humans: SUMO1 to SUMO4 (19). SUMO1 modifies proteins as a monomer, while SUMO2 and SUMO3 can form chains. It is not clear whether SUMO4 can modify proteins. SUMO2 and SUMO3 are 95% homologous in sequence, and no current antibodies can distinguish between the two isoforms (20). Other SUMOylation inhibitors have been developed that target different members of the SUMO ligase pathway for chemotherapy (21). The lack of toxicity of benzotriazine analogues, together with our previously published RNA-Seq results, suggest that these compounds do not affect general SUMOylation but specifically target STAT proteins (8).

STATs are a family of seven proteins (STAT1, STAT2, STAT3, STAT4, STAT5A, STAT5B, and STAT6) that share between 30 to 40% amino acid identity and are master transcriptional regulators that control cellular fate in response to the extracellular environment. STAT function is required for cellular proliferation and survival, antiviral responses, inflammation, cell motility, and others (for a review of STAT functions, see reference 22). In addition to STAT5, other STAT proteins have been shown to be regulated by SUMOylation. SUMOylation of STAT1 reduces gamma interferon (IFN-γ) and IFN-β induction of interferon-stimulated genes, negatively regulating the antiviral response (23–25); STAT3 is also regulated by SUMOylation in a mechanism that involves SUMO2/3 (26, 27). Finally, STAT4 expression levels have been shown to be regulated by the E3 SUMO ligase protein inhibitor of activated STATx (PIASx) (28). STATs play also a major role in regulating CD8 T cell responses (29). In fact, expression of a constitutively active form of STAT5 in murine CD8 T cells promotes the expression of genes controlling effector molecules, proliferation, and tissue homing, as well as transcription factors required for CD8 T cell function, such as T-bet and Eomes (30–32). STAT proteins have also been shown to play important roles in NK function (for a review, see reference 33). These studies suggest that targeting STAT SUMOylation may also improve antiviral responses, as well as the cytolytic function of CD8 T cells and NK cells. Our results suggest that targeting STAT SUMOylation may also have immunomodulatory properties in CD8 T and NK cells. It will be interesting to further address whether benzotriazine analogues may also have antiviral and immunoenhancing properties in CD8 T cells and NK cells.

Our initial *in vivo* toxicity studies suggest that benzotriazine analogues present low acute toxicity. Further studies are warranted to evaluate the long-term toxicity, pharmacodynamics, pharmacokinetics, and efficacy of these compounds *in vivo*. In conclusion, targeting STAT SUMOylation may represent a novel therapeutic strategy, and further development of these compounds represents a compelling step toward fully evaluating their potential application for HIV cure approaches.

## MATERIALS AND METHODS

### Reagents.

The following reagents were obtained through the NIH AIDS Reagent Program, Division of AIDS, NIAID, NIH: nelfinavir and human rIL-2 were obtained from Maurice Gately, Hoffmann-La Roche, Inc. (34), and pNL4-3 were obtained from Malcolm Martin (35). CD3/CD28 Dynabeads were obtained from Thermo Fisher Scientific. pLX304-STAT5A (catalog no. 17568) and pLX304-STAT5B (catalog no. 17598) were obtained from the DNASU plasmid repository. Benzotriazine, benzotriazole, and other chemicals were purchased from various commercial sources (see Data Set S1 in the supplemental material). All the analogues were prepared at 1 M in DMSO. When needed, intermediate dilutions were made in RPMI medium supplemented with 10% fetal bovine serum (FBS), 1% l-glucose, and penicillin-streptomycin. Final concentration of DMSO in each experiment was kept below 0.5%.

### 293FT-STAT5A cell line.

A bicistronic lentiviral vector derived from pFIN-EF1-GFP-2A-mCherry-WPRE was engineered to express STAT5A in place of green fluorescent protein (GFP) (36). The resulting lentiviral vector, pFIN-EF1-STAT5A-2A-mCherry-WPRE, encodes a fusion of STAT5A and mCherry, the expression of which is driven by the elongation factor 1 (EF1) promoter. The presence of the intervening 2A peptide from porcine teschovirus-1 leads to ribosomal skipping and equimolar production of STAT5A and mCherry (36). After transduction of 293FT cells, mCherry-positive cells were sorted using flow cytometry-activated cell sorting, and single clones were generated by limited dilution.

### HEK-Blue IL-2 cells.

HEK-Blue IL-2 cells were obtained from Invivogen. HEK293 cells were transformed with adenovirus 5 DNA and transfected with hIL-2Rα, hIL2-Rβ, hIL-2Rγ, hJAK3, and hSTAT5 genes. The cells were also transfected with a SEAP reporter gene under the control of the IFN-β promoter fused with STAT5 binding sites. Cells were maintained in Dulbecco modified Eagle medium supplemented with 10% (vol/vol) heat-inactivated FBS, 1% penicillin, 1% streptomycin, 1× HEK-Blue CLR selection cocktail, and 1 μg/ml puromycin.

HEK-Blue IL-2 cells were plated at 50,000 cells/well in a 96-well flat-bottom plate for 24 h prior to treatment to facilitate adherence. Cells were treated in sextuplet for each concentration for 24 h. A positive control of 1 ng/ml hIL-2 and a negative control of 10 ng/ml hTGF-β were used. After 24 h of treatment, plates were spun down at 15,000 × *g* for 5 min before 20 μl of each well was transferred to a fresh 96-well flat-bottom plate. Then, 180 μl of prepared Quanti-Blue solution was added to each well, and plates were incubated at 37°C for 2 h. SEAP levels were determined using a spectrophotometer at 640 nm. To evaluate toxicity, 50 μl of each well was transferred to a fresh 96-well flat-bottom plate. Next, 50 μl of prepared CytoTox 96 reagent was added to each well, and the plates were incubated at room temperature for 30 min in the dark. Finally, 50 μl of stop solution was added to each well, and the absorbance was recorded using a spectrophotometer at 490 nm.

### Generation of latently infected cultured T_CM_.

Naive CD4^+^ T cells were isolated via negative selection from PBMCs obtained from healthy donors. Cultured T_CM_ cells were generated and infected as previously described (12, 37).

### Flow cytometry analysis.

To assess surface expression of CD69, 1 × 10^5^ cells were stained first with 0.1 μl of viability dye (eBioscience fixable viability dye eFluor 450) in 100 μl of phosphate-buffered saline (PBS) for 10 min at 4°C. The cells were then stained with 0.5 μl of antiCD69-APC (CD69 monoclonal antibody [CH/4]; Thermo Fisher Scientific) in 100 μl of PBS plus 3% FBS for 30 min at 4°C.

To assess the intracellular p24Gag expression, 1 × 10^5^ cells were fixed, permeabilized, and stained as previously described (12). In all experiments, HIV-1 p24Gag-negative staining regions were set with uninfected cells treated in parallel.

To check NK cell and T cell activation, PBMCs were collected from wells and washed with PBS plus 3% FBS. Cells were stained with Live/Dead fixable aqua stain (Invitrogen) and washed, and the following anti-human antibodies were used for staining: CD3-BV786 (clone SP34-2; catalog no. 563800 [Becton Dickinson]), CD4-Pacific Blue (clone RPA-T4; catalog no. 558116 [Becton Dickinson]), CD8-AlexaFluor700 (clone OKT8; catalog no. 56-008-42 [eBioscience]), CD56-BV605 (clone HCD56; catalog no. 318334 [BioLegend]), and CD69-APCCy7 (clone FN50; catalog no. 310914 [BioLegend]).

Cells were analyzed on a BD LSR Fortessa X20 flow cytometer with FACSDiva software (Becton Dickinson, Mountain View, CA) and analyzed using FlowJo (TreeStar, Inc., Ashland, OR).

### Western blotting.

To analyze STAT5 phosphorylation, cells were lysed with NETN extract buffer containing 100 mM NaCl, 20 mM Tris-Cl (pH 8), 0.5 mM EDTA, 0.5% Nonidet P-40, protease inhibitor cocktail (cOmplete; Roche), and phosphatase inhibitor cocktail (phosSTOP; Roche) for 30 min on ice. Lysates were cleared by centrifugation at 12,000 rpm for 10 min at 4°C. Proteins were visualized by SDS-PAGE. Western blotting was performed according to standard protocols. The following antibodies were used at the following concentrations: anti-STAT5 at a 1:1,000 dilution (clone D2O6Y; Cell Signaling Technology, Danvers, MA), anti-phospho STAT5 (Tyr694) at a 1:1,000 dilution (clone C11C5; Cell Signaling Technology), and anti-β-actin antibody at a 1:10,000 dilution (clone AC-15; Sigma-Aldrich, St. Louis, MO). Secondary anti-rabbit and secondary anti-mouse antibodies (Jackson ImmunoResearch, catalog no. 111-035-046 and 115-035-146, respectively) were used at a 1:10,000 dilution.

### REVEAL assay.

Human primary PBMCs were obtained via peripheral phlebotomy as described above and resting CD4^+^ T cells were isolated using a magnetic bead negative-selection kit from StemCell Technologies according to the manufacturer’s instructions. *Ex vivo* cell culture assays with resting CD4^+^ T cells from HIV-1-infected aviremic participants were performed as previously described (38, 39). Briefly, cells were cultured at a concentration of four million cells/ml of culture medium per condition. Cells were exposed to IL-15 (100 ng/ml), followed by the addition of benzotriazine analogues (100 μM), and then cultured for 48 h. Cells then underwent centrifugation, and cell-associated RNA was extracted. RNA isolation and purification were performed using TRIzol RNA isolation according to the manufacturer’s instructions. For the detection of HIV-1 mRNA transcripts, qPCR was performed on cell-associated RNA isolated from cultured cells, as described previously (38, 39).

### Digital p24 ELISA.

A total of 10^7^ PBMCs from ART-suppressed PLWH were cultured for 4 days in a 12-well plate containing 3 ml of RPMI 1640 medium (10% FBS, 1% penicillin-streptomycin, 1% l-glutamine) with 1 μM raltegravir and 0.5 μM nelfinavir, either in medium control (untreated), 50 ng/ml PMA plus 1 μM ionomycin, or 100 μM HODHBT. Unidentified samples were sent to Quanterix (Lexington, MA) for analysis of p24 using a SIMOA assay (18, 40). Samples were analyzed in duplicate.

### Cytokine analysis.

Culture supernatants were collected from each well and stored at –80°C until ready for analysis. Thirteen cytokines were measured by using a LEGENDplex human Th cytokine panel kit according to the manufacturer’s protocol (BioLegend, San Diego, CA). The following cytokines were measured using this test: IL-2, IL-4, IL-5, IL-6, IL-9, IL-10, IL-17A, IL-17F, IL-21, and IL-22.

To analyze IL-6 in mouse sera, 200 μl of blood was collected in small tubes. Blood was undisturbed at room temperature for 15 to 30 min to clot. Each clot was pelleted by centrifuging at 1,000 to 2,000 × *g* for 10 min in a refrigerated centrifuge. The serum was carefully collected with a pipette tip. IL-6 was measured with a LEGENDplex mouse IL-6 kit according to the manufacturer’s protocol (BioLegend).

### Animal studies.

Animal studies were performed according to an active Institutional Animal Care and Use Committee protocol (A377) approved by George Washington University.

Male and female C56BL/6 mice (6 to 9 weeks old) were obtained from The Jackson Laboratory. Compounds were first resuspended in DMSO at 1 M and then diluted to the appropriate concentration in Dulbecco PBS. The total DMSO concentration injected into each mouse was 4%. Five mice per group were injected with the compounds at 0, 1, 10, 50, and 100 mg/kg via the s.c. route or at 0, 1, 10, and 50 mg/kg by the i.p. route. Mice were monitored for 8 h after administration for signs of toxicity and weighed daily for 14 days. After 14 days, the mice were euthanized for necropsy.

### Participant involvement.

**(i) Cells from uninfected blood donors.** Blood donors 18 years and older served as blood donors. Written informed consent was obtained from all donors. These studies are covered under the Institutional Review Board protocol 67637 approved by the University of Utah Institutional Review Board. Alternatively, blood was obtained from the Gulf Coast Regional Blood Center (Houston, TX).

**(ii) Cells from infected HIV-1^+^ donors (REVEAL assay).** Aviremic HIV-1-infected patients on ART were recruited for phlebotomy according to an approved and active institutional review board protocol (IRB_0058246) at the University of Utah, as described previously (38). Inclusion criteria for this study required viral suppression (<50 HIV-1 RNA copies/ml) for a minimum of 6 months, ART initiation during chronic HIV-1 infection (>6 months since seroconversion), and compliance with a stable ART regimen for a minimum of 12 months per participant and provider report. Informed consent and phlebotomy were performed in the Center for Clinical and Translational Science Clinical Services Core at the University of Utah Medical Center.

**(iii) Cells from aviremic participants.** Cells from aviremic participants were obtained through the Reservoir Characterization Section of the BELIEVE collaborative (IRB 021750). Secondary use of the samples was approved through George Washington University institutional review boards. All subjects were adults and gave informed consent.

### Statistics.

Two-tailed paired-sample *t* test analysis or a Wilcoxon matched-pair signed-rank test was used to calculate *P* values. When appropriate, column statistics and *P* values were calculated. *P* values were calculated using Prism 5 for Mac OS X software (GraphPad Software, Inc., La Jolla, CA).

## Supplementary Material

Supplemental file 1

Supplemental file 2
