# Supplementary material for: Structure-Activity Relationship Analysis of Benzotriazine Analogues as HIV-1 Latency-Reversing Agents
Source: Antimicrob Agents Chemother. 2020 Jul 22;64(8):e00888-20. doi: 10.1128/AAC.00888-20 (PMC7526807; doi:10.1128/AAC.00888-20)
Supplement: Supplemental file 1 [file AAC.00888-20-s0001.pdf]

Supplemental Information

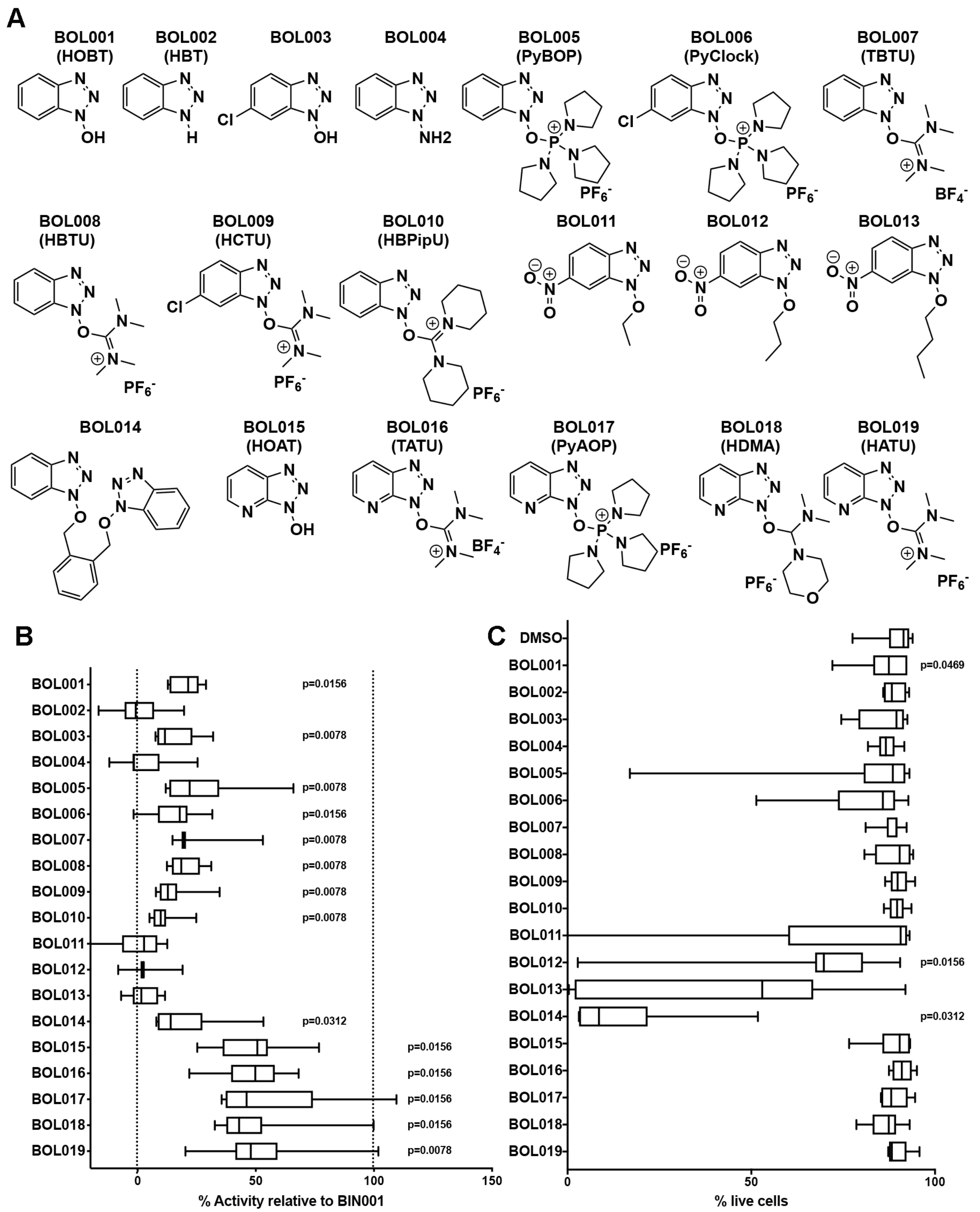

Sup Figure 1. Screening for biological activity of benzotriazole analogues. A. Structure of 21 commercially available benzotriazole analogues. **B.** Analysis of activity of

the different analogues relative to BIN001 (HODHBt), the derivative with the highest LRA activity, in 9 different donors. Error bars indicate maximum and minimum and Wilcoxon signed rank test was used to calculate p values. **C.** Analysis of toxicity of the different analogues. Error bars indicate maximum and minimum. Nonparametric Wilcoxon matched-pairs signed rank test relative to DMSO control was used to calculate p values.

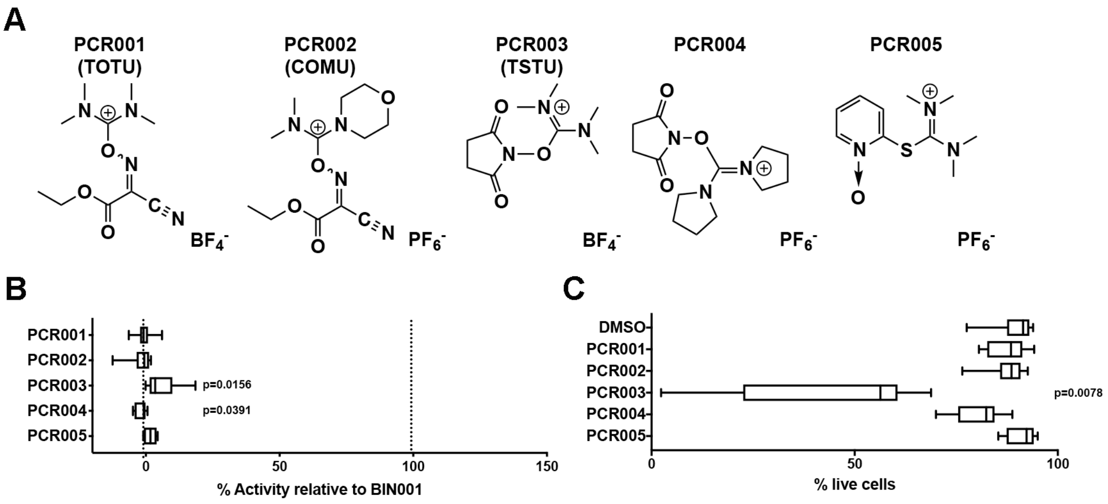

Sup Figure 2. Screening for biological activity of peptide couple reagents. A. Structure of 5 commercially available protein coupling reagents. **B.** Analysis of activity of the different analogues relative to BIN001 (HODHBt), the derivative with the highest LRA activity, in 9 different donors. Error bars indicate maximum and minimum and Wilcoxon signed rank test was used to calculate p values. **C.** Analysis of toxicity of the different analogues. Error bars indicate maximum and minimum. Nonparametric Wilcoxon matched-pairs signed rank test relative to DMSO control was used to calculate p values.

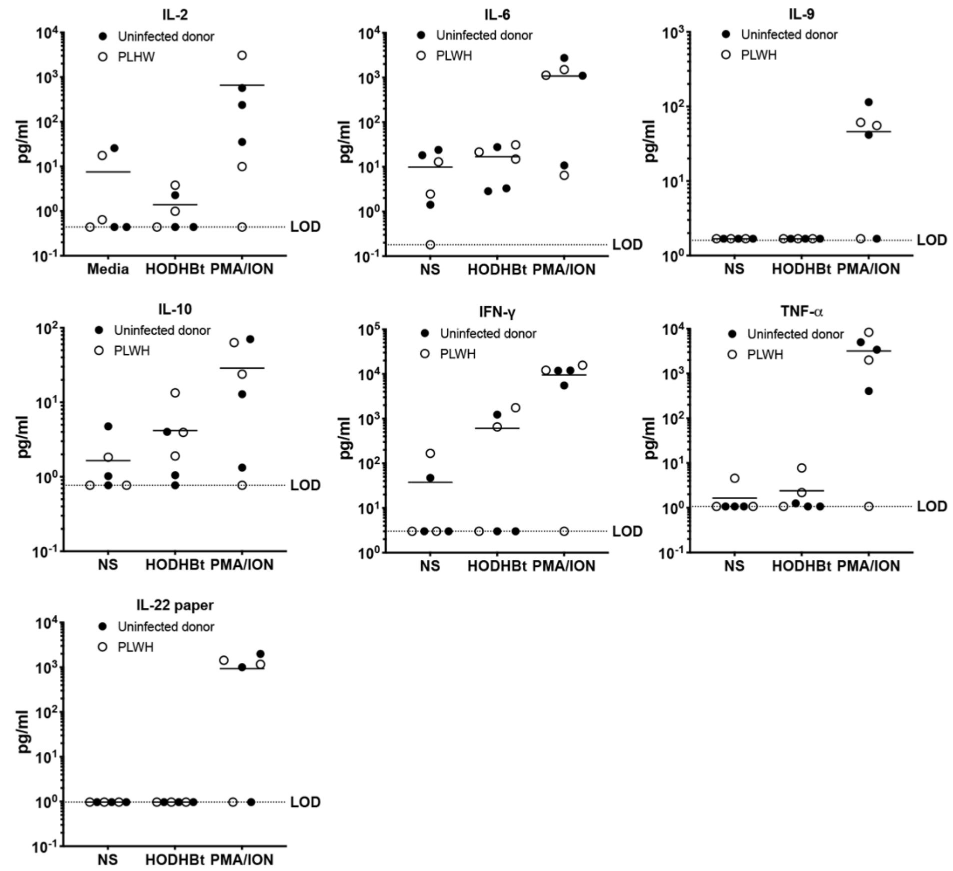

Sup Figure 3. Levels of secreted cytokines induced by HODHBt. Levels of several pro- and anti-inflammatory cytokines in the supernatant of PBMCs from 3 HIV-negative donors (uninfected donor) or 3 ART-suppressed PLWH 48 hours after treatment with either HODHBt or PMA plus Ionomycin (PMA/IONO). LOD: limit of detection.

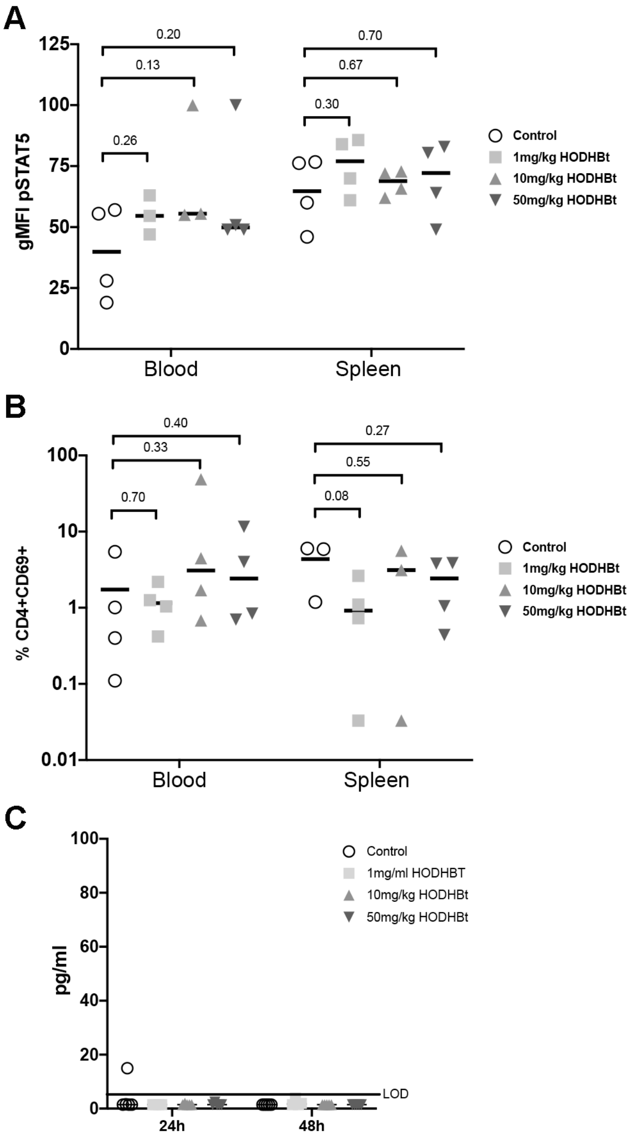

Sup Figure 4. Pharmacodynamics of HODHBt. A. Levels of pSTAT5 were measured in either PBMCs isolated from blood or total splenocytes 48 hours post intraperitoneal injection of HODHBt at the concentrations indicated. **B.** Levels of CD69 measured in CD4 T cells from blood or total splenocytes 48 hours post intra peritoneal injection of HODHBt at the concentrations indicated. Unpaired t-test was used to calculate p values. **C.** Levels of IL-6 in serum isolated 24 and 48 hours post intra peritoneal injection of HODHBt at the concentrations indicated. LOD: limit of detection.
